# Supplementary material for: Schiff Base Compounds as Fluorescent Probes for the Highly Sensitive and Selective Detection of Al3+ Ions
Source: Molecules. 2023 Mar 30;28(7):3090. doi: 10.3390/molecules28073090 (PMC10096070; doi:10.3390/molecules28073090)
Supplement: Supplementary file 1 [file molecules-28-03090-s001.zip › molecules-2284735-supplementary.pdf]

Supplementary Materials

Schiff Base Compounds as Fluorescent Probes for the Highly Sensitive and Selective Detection of Al3+ Ions

Yanling Pang [1], Desu Meng [1], Jian Liu [2,*], Shengxia Duan [1,*], Jingru Fan [1], Longyu Gao [1] and Xinshu Long [1]

1 Department of Chemistry and Engineering, Heze University, Heze 274500, China

2 College of Agriculture and Bioengineering, Heze University, Heze 274000, China

* Correspondence: liujian61@hezeu.edu.cn (J.L.); duanshengxia@hezeu.edu.cn (S.D.)

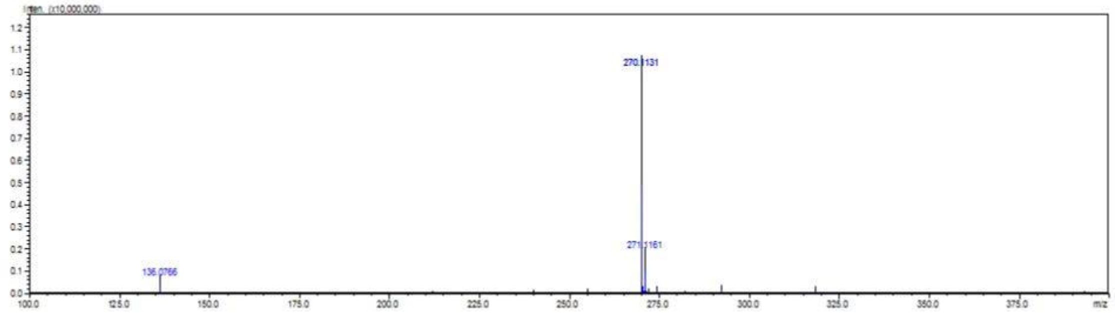

Figure S1 HRMS spectrum of L.

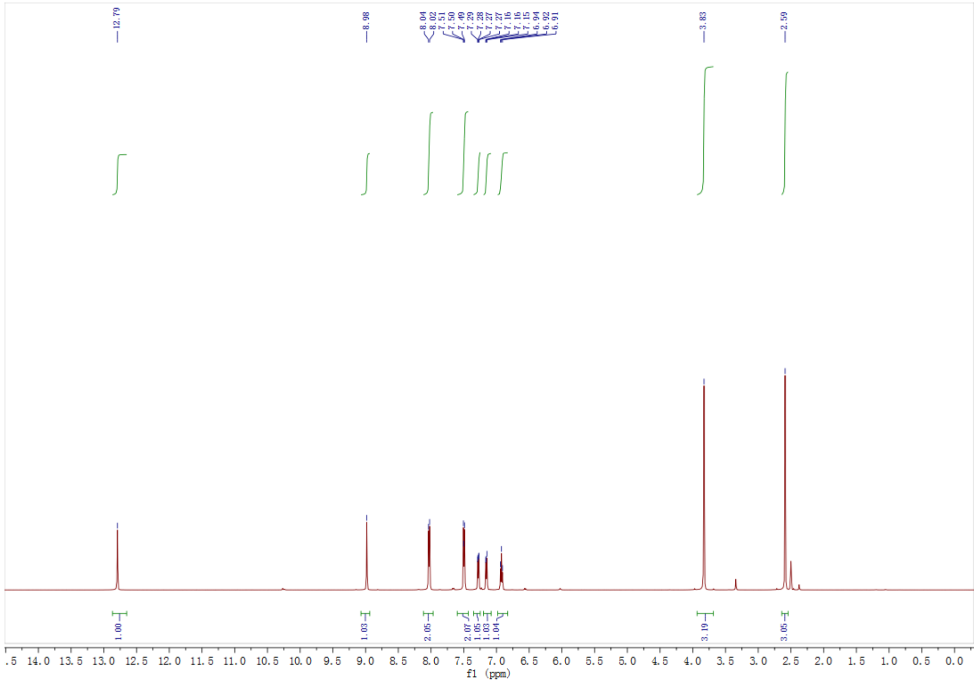

Figure S2 ^{1}H NMR spectrum of L.

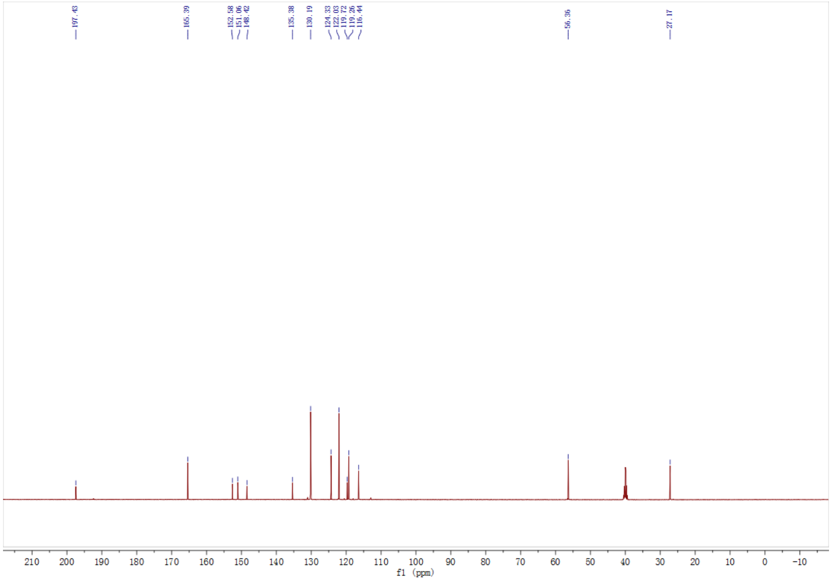

Figure S3 ^{13}C NMR spectrum of L.

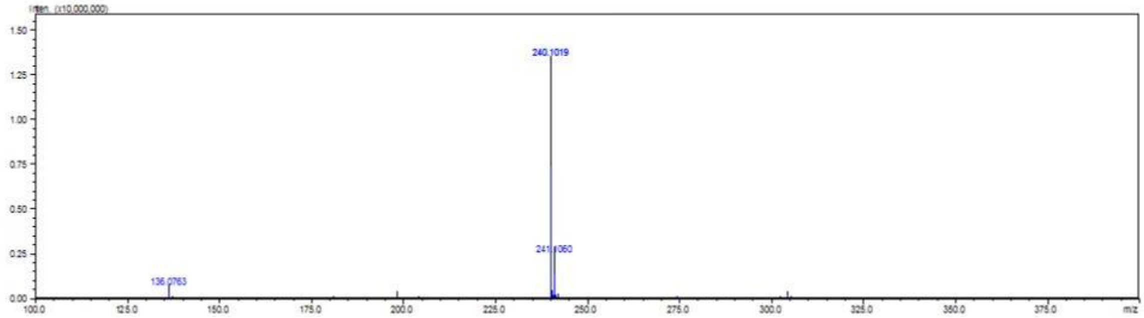

Figure S4 HRMS spectrum of S.

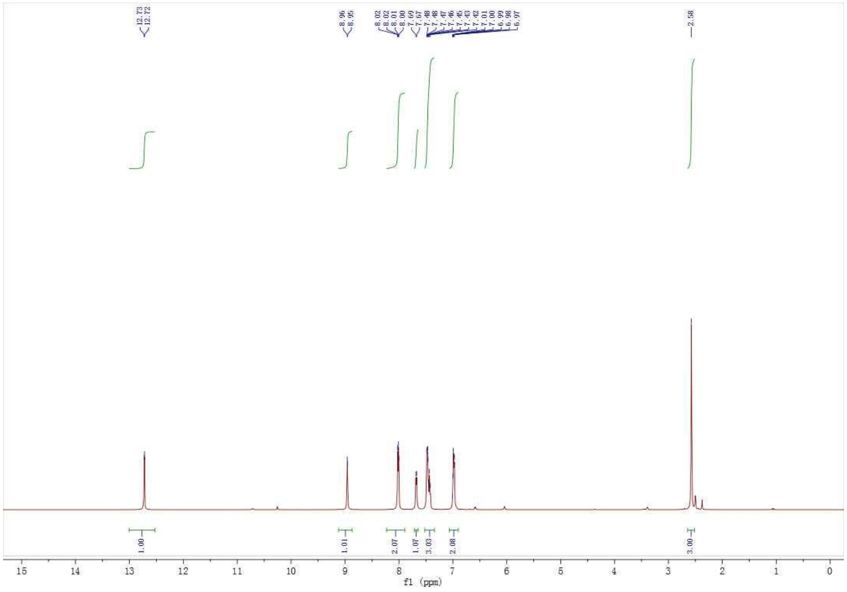

Figure S5 ^{1}H NMR spectrum of S.

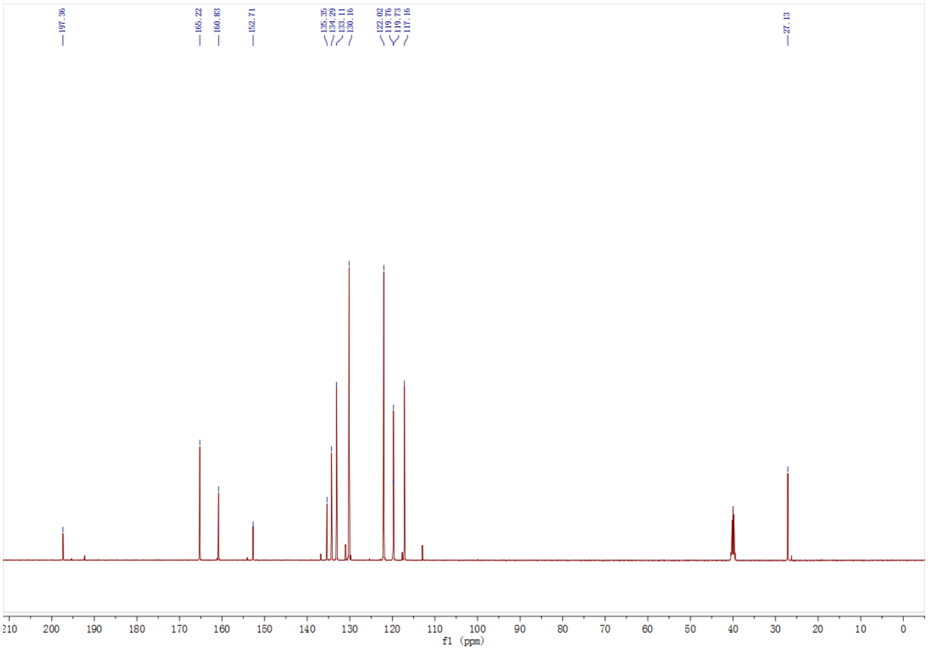

Figure S6 ^{13}C NMR spectrum of S.
